# Resuscitation Using Liposomal Vasopressin in an Animal Model of Uncontrolled Hemorrhagic Shock

**DOI:** 10.1371/journal.pone.0130655

**Published:** 2015-07-08

**Authors:** Meng-Tse Gabriel Lee, Hsuan-Mao Wang, Ja-An Annie Ho, Nien-Chu Fan, Ya-Lin Yang, Chien-Chang Lee, Shyr-Chyr Chen

**Affiliations:** 1 Department of Emergency Medicine, National Taiwan University Hospital, Taipei, Taiwan; 2 Department of Biochemical Science and Technology, National Taiwan University, Taipei, Taiwan; 3 Department of Emergency Medicine, National Taiwan University Hospital Yunlin Branch, Douliou, Taiwan; 4 Department of General Medicine, National Taiwan University Hospital Yunlin Branch, Douliou, Taiwan; Max-Delbrück Center for Molecular Medicine (MDC), GERMANY

## Abstract

**Background:**

Current research suggests that administration of vasopressin to patients with uncontrolled hemorrhagic shock (UHS) can avoid the detrimental effects associated with aggressive fluid resuscitation. However, vasopressin has a short half-life of 10~35 minutes in *in vivo* use and precludes its use in the pre-hospital setting. To increase the half-life of vasopressin, we proposed to synthesize liposome-encapsulated vasopressin and test it in a rat model of UHS.

**Methods:**

The film hydration method was used to prepare liposomal vasopressin consisting of: Dipalmitoylphosphatidylcholine, cholesterol, and dipalmitoyl phosphatidylethanolamine (20:20:1 mole ratio). 42 rats were subjected to UHS and randomly received 5 different treatments (vasopressin, liposomal vasopressin, lactate ringer (LR), liposome only and sham). Outcome of UHS were measured using 4 common prognostic tests: mean arterial pressure (MAP), serum lactate level, inflammatory profile and pulmonary edema.

**Results:**

The dynamic light scattering results confirmed that we had prepared a successful liposomal vasopressin complex. Comparing the serum vasopressin concentration of liposomal vasopressin and vasopressin treated animals by ELISA, we found that the concentration of vasopressin for the liposomal vasopressin treated group is higher at 60 minutes. However, there was no significant difference between the MAP profile of rats treated with vasopressin and liposomal vasopressin in UHS. We also observed that animals treated with liposomal vasopressin performed indifferently to vasopressin treated rats in serum lactate level, inflammatory profile and edema profile. For most of our assays, the liposome only control behaves similarly to LR resuscitation in UHS rats.

**Conclusion:**

We have synthesized a liposomal vasopressin complex that can prolong the serum concentration of vasopressin in a rat model of UHS. Although UHS rats treated with either liposomal vasopressin or vasopressin showed no statistical differences, it would be worthwhile to repeat the experiments with different liposomal compositions.

## Introduction

Millions of people died from traumatic injury every year [1, 2]. It has been estimated that about one-third of those with severe traumatic injuries suffer from trauma-associated hemorrhagic shock [3]. Hemorrhagic shock refers to a life-threatening condition where more than one-fifth of the body’s blood is lost and there is inadequate delivery of oxygen and nutrients to body organs. Mortality can result directly from the massive blood loss or indirectly due to multiple organ failure. In particular, loss of gastrointestinal, renal, hepatic, and pulmonary function is frequent after uncontrolled hemorrhagic shock [4, 5].

The traditional treatment for uncontrolled hemorrhagic shock (UHS) is rapid fluid infusion to restore blood pressure. However, recent animal and clinical studies have revealed that attempting to achieve normal blood pressure by early aggressive fluid resuscitation for UHS increased mortality instead [6–10]. This is because early aggressive fluid resuscitation can cause dilution of clotting factors, decreased blood viscosity, and dislodgement of blood clots [11–14]. The early administration of vasopressin has been found to maintain tissue perfusion and can avoid the detrimental effects associated with aggressive fluid resuscitation [15, 16].

Vasopressin is a natural antidiuretic hormone that functions to retain water in the body and to constrict blood vessels. Unfortunately, vasopressin has limited clinical application due to its short *in vivo* half-life of 10~35 minutes [17–19]. Patients with refractive shock may require continuous infusion of vasopressin, and precludes its use in the pre-hospital setting where severe hemorrhagic shock occurs.

Liposomes have been shown to improve delivery of peptide drugs by protecting encapsulated peptide from enzymatic degradation and act as a “microreservoir” to release the peptide slowly at the site of injection [20–22]. Hence, we hypothesized that liposome-encapsulated vasopressin will have a longer half-life than vasopressin and therefore give a better outcome in UHS. Outcome in UHS were measured using common prognostic tests: mean arterial pressure (MAP), serum lactate level, inflammatory profile and pulmonary edema.

## Methods

### Liposome preparation

The film hydration method, which had been described previously by Ho’s group was used to prepare liposomal vasopressin.[23–25] Briefly, the lipid mixture consisting of DPPC (Dipalmitoylphosphatidylcholine), cholesterol, and DPPE (dipalmitoyl phosphatidylethanolamine) (20:20:1 mole ratio) was dissolved in chloroform (4 mL). The mixture is then sonicated for 3 minutes. After that, we used reduced pressure to evaporate the organic solvent, leaving a thin lipid film on the round-bottom flask. Vasopressin solution at a conc. of 0.5 U/mL was added to dissolve the lipid film. This is followed by sonication for 3 more minutes at 45°C. The final sample is then stored at -20 degree until usage.

### Dynamic light scattering

The size and polydispersity of liposomal vasopressin were measured using dynamic light scattering (DLS) techniques. The DLS measurements were performed with a Malvern Zetasizer Nano ZS (Malvern, Herrenberg, Germany) equipped with a 633-nm He-Ne laser and operating at an angle of 173°. The measurements were made at a position of 4.65 mm from the cuvette wall with an automatic attenuator and at a controlled temperature of 25°C. The intensity size distribution, the peak diameter and the polydispersity index (PdI) of liposomal vasopressin were obtained from the autocorrelation function using the “general purpose mode”.

### Experimental animals

The National Taiwan University Hospital Animal Protocol Review Committee has approved the protocols conducted in this research (approval ID: 20060318). Wistar male rats (N = 42; weight 300–400 g) were fasted overnight but allowed water ad libitum before inducing hemorrhagic shock. To alleviate pain and suffering, the rats were initially anesthetized with 2% isoflurane (mixed with pure oxygen) until they stopped making a movement. Then the rats were continuously maintained with isoflurane (1%, mixed with pure oxygen) throughout the 135-min observation period. We used an aseptic technique to isolate and cannulate the left femoral artery and vein (PE-50, Becton Dickinson, Sparks, MD). The venous catheter was used for fluid and vasopressor infusion, while the arterial catheter was connected to a pressure transducer (BP-2 Digital Blood Pressure Monitor; Columbus Instruments, Columbus, Ohio). We used the pressure transducer for blood withdrawal, and continuous mean arterial pressure (MAP) monitoring. In addition, the health and condition of the animals were closely examined at 7 time points (0min, 15 mins, 30 mins, 60mins, 90mins, 150 mins and 72 hours).

### Rat model of uncontrolled hemorrhagic shock

We used a modified UHS model developed by Capone and adopted by other researcher such as Poloujadoff [26–28]. Our experimental model was designed to simulate the life-support chain of emergency medical service for trauma, which consists of three phases. First, UHS phase I, represents prehospital care, span from 0 to 75 minutes. We conducted an initial 15-minute volume-controlled hemorrhage (3 mL/100 g). After the controlled hemorrhage, we carried out uncontrolled hemorrhage by 80% tail amputation. Care was taken to make sure all the 42 animals were treated in the same way. In our experiment, 42 rats were randomly divided to five treatment groups (vasopressin, liposomal vasopressin, lactate ringer, liposome only and sham), and injected with different drugs at 30 minutes. All the different drugs are diluted to a final volume of 1ml using LR solution. The first group of animals, vasopressin treated group (N = 11), received intravenous 0.8 U/kg vasopressin (Pitressin, Parke-Davis). The second group of animals, liposomal vasopressin treated group (N = 13), received intravenous 0.8 U/kg liposomal vasopressin. The third group of animals, Lactate Ringer’s solution (LR) treated group (N = 10), received an equal volume of LR solution. The fourth group of animals (N = 4), liposome only group, received an equal volume of liposome solution mixed with LR and did not receive any vasopressors. The final group of animals, sham group (N = 4), underwent exactly the same anesthetic regimen and monitoring as the study animals but without hemorrhagic shock, tail amputation, and fluid resuscitation.

The second phase, which is the resuscitation phase, represents “hospital” care. This phase start from 75 minutes and end at 135 minutes. Normotension was restored for all experimental groups by infusion of lactated Ringer’s (LR) solution that is 2X the volume of the shed blood, followed by the shed blood. Hence, the total infused volume was 3X the volume of shed blood. The final phase, phase III, the observation phase, lasted from 135 mins to 72 hrs.

### Determination of vasopressin, lactates and cytokines

At 4 different time points (30, 60, 90, and 150 min), 1.5ml blood samples were obtained for determination of serum levels of vasopressin, lactates and cytokines. We determined the serum concentration of vasopressin by a commercially available ELISA with specific monoclonal antibodies (Cayman Chemical Company, MI, USA). Serum levels of lactates were determined using a Stat Profile Critical Care Xpress machine (Nova Biomedical, Waltham, MA). The level of cytokine IL-6 and TNF-α were determined by a commercially available ELISA with specific monoclonal antibodies (Biosource International, USA), and the reliability of these cytokine kits had been shown in previous studies.[29, 30] All experiments were performed according to the manufacturer’s instructions.

### Pulmonary edema

At the end of the 72 hours from UHS, the health and mortality of animals were checked before carrying out pulmonary edema studies. The rats were anesthetized by isoflurane (1–2%, mixed with pure oxygen) before carrying out medial sternotomy. We euthanized the rats by cutting open the ascending aorta directly. The lungs were then removed from the thoracic cavity. After dissecting the inferior third of the left lung, we weighed it before and after drying. Drying is carrying out using oven at 90°C for 24 hours. The extent of edema was calculated by the ratio of the weight before and after drying.

### Statistics

We used a statistical analysis similar to our previously published report.[27] The statistical analyses were carried out using SPSS statistical package (version 21.0, IBM SPSS Statistics, IBM Corporation, Armonk, NY). All graphical points represent a single comparison of the average over time, unless stated otherwise. We presented descriptive values as mean ± standard deviation for continuous variables and absolute count with relative frequencies for categorical variables. Repeated measure analysis of variance (rmANOVA) with a grouping factor was used to analyze differences of hemodynamic parameters and serum levels of cytokine. The repeated measure was time, and treatment strategy was used as the grouping factor. We checked the basic assumptions of rmANOVA to make sure they were not grossly violated. Since the group by time results may be solely due to the sham group differing from the other three, comparison was limited to the treatment groups of clinical interest. Tukey’s method was used to conduct post hoc analysis. The mean difference between indicated treatment groups was considered to be significant at 0.05.

## Results

### Characterization of liposome encapsulated vasopressin

**Fig 1:**
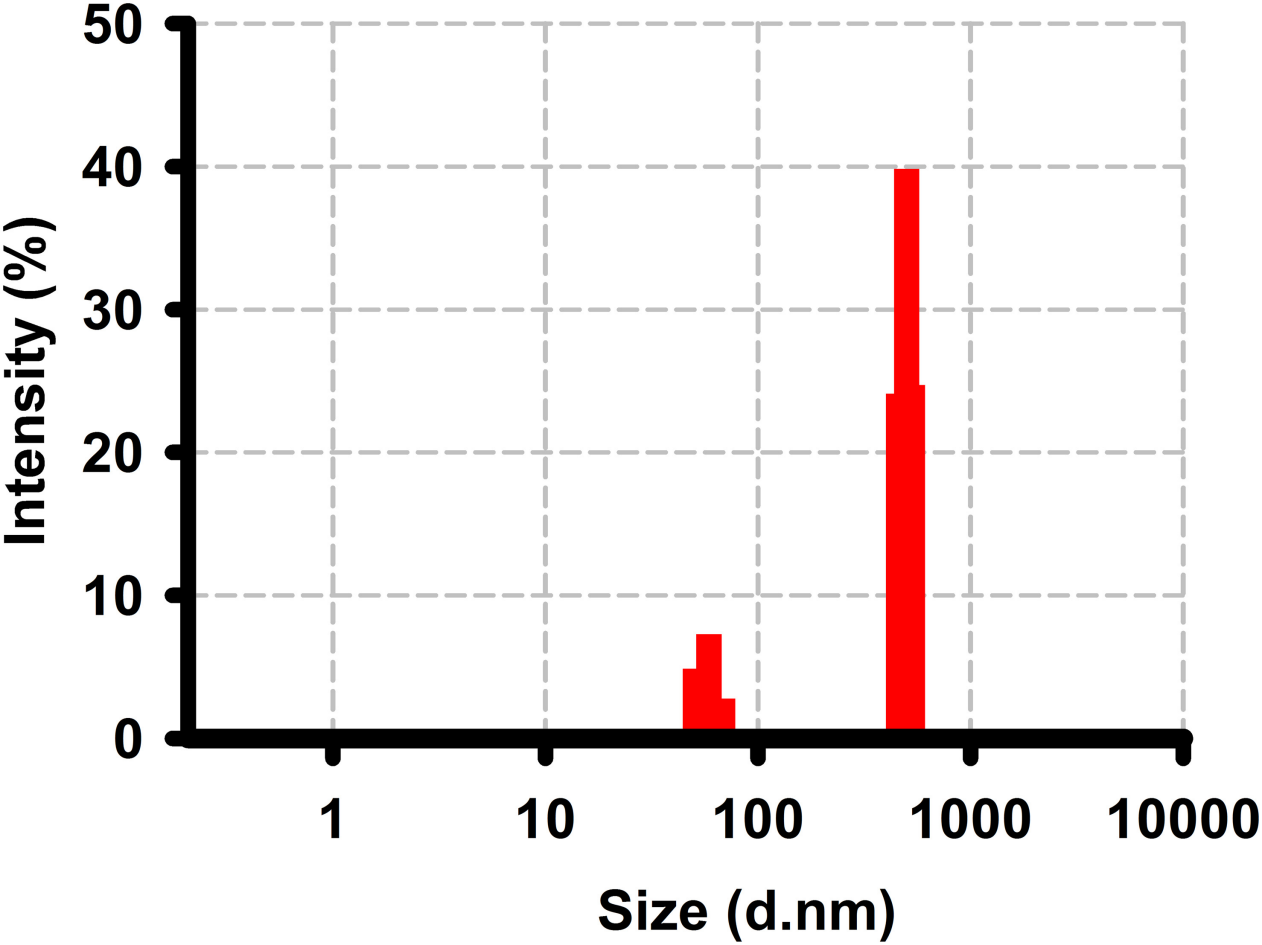
Dynamic light scattering of the liposomal vasopressin preparation.

Fig 1 shows the size and polydispersity of a representative liposomal vasopressin preparation. The resulting liposome suspension shows an average vesicle diameter of 535nM. Based on past peptide encapsulation experience, at least 10% encapsulation efficiency can be achieved. We also determined that the liposomal vasopressin complex was stable using our storage method. The physical stability of liposomal vasopressin was evaluated by physical appearance and the mean diameter of liposomes.

The *in vitro* stability of liposomal complex may not correlate directly with *in vivo* stability of the liposomal complex. To gain insights into whether the encapsulated vasopressin can increase the serum half-life of vasopressin, we carried out a vasopressin ELISA experiment at different time points (S1 Fig). The liposomal vasopressin that is administered contains a mixture of encapsulated and un-encapsulated drug fraction, as it is not possible to obtain a pure encapsulated fraction with the present setup. Comparing liposomal vasopressin and vasopressin treated animal at 60 minutes, we found that there is indeed 2 fold higher serum concentration of vasopressin for the liposomal vasopressin treated group (Avg: 1904 VS 899 pg/ml). However, at 90 and 150 minutes, there is no significant difference in serum concentration of vasopressin in liposomal vasopressin, vasopressin, LR and liposome only treated groups.

### Mean arterial pressure (MAP)

**Fig 2:**
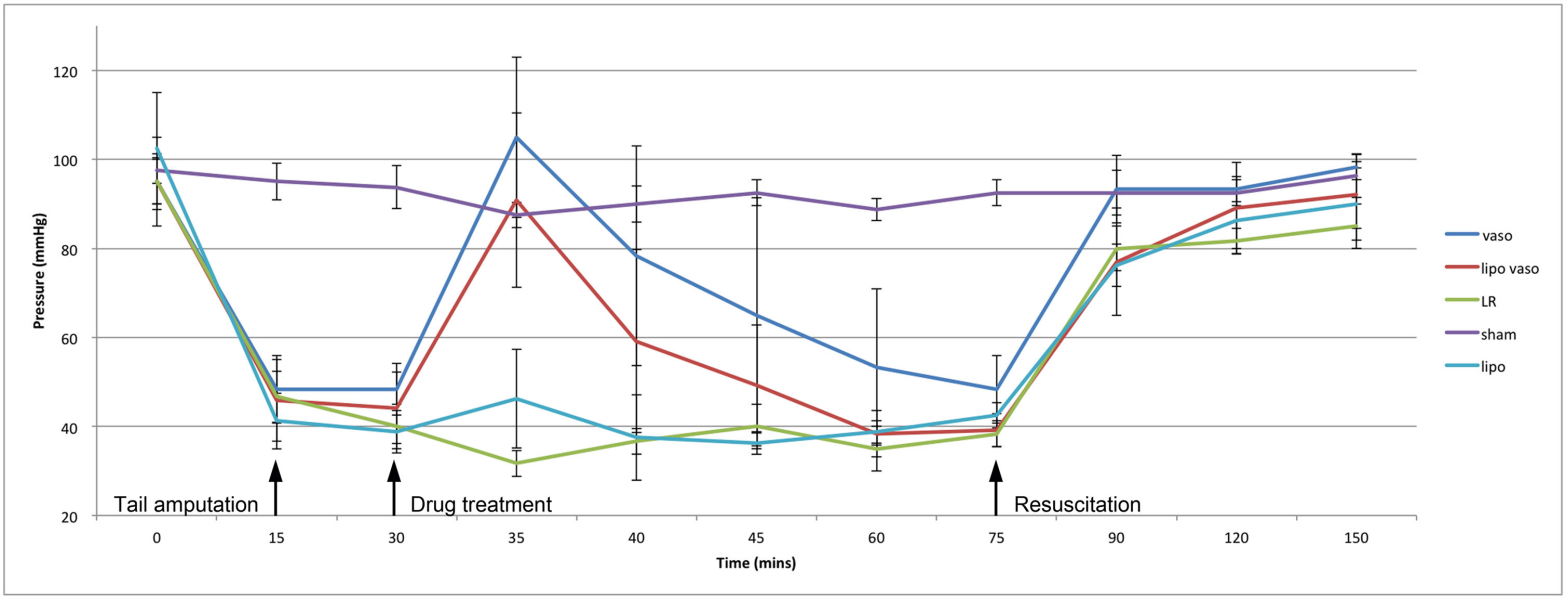
Mean arterial pressure for the five treatment groups over time. Vaso group refers to rats treated with vasopressin. Lipo vaso group refers to rats treated with liposomal vasopressin. LR group refers to rats treated with lactated ringer solution without any drug. Sham group refers to rats not undergoing any treatment. Lipo group refers to rats treated with liposome only.

Serial measurements of mean (±SD) arterial pressure for animals with different drug treatments were plotted against time. Vasopressin’s (Fig 2, blue line) administration resulted in an increased in MAP as expected. The effect of vasopressin on the MAP could not be sustained after 5 minutes, and fall gradually to the level observed in Lactate Ringer’s solution (LR) treated group. Unexpectedly, the MAP profile of vasopressin and liposomal vasopressin (red line) behaved similarly, and statistical analysis showed that there was no significant difference. To rule out the possibility that liposome (Lipo) can cause an adverse effect to the blood pressure; the MAP profile of animals treated with either Lipo (cyan line) or LR (green line) was compared. There was no significant difference in the MAP profile of LR and Lipo treated group.

### Serum lactate levels

**Fig 3:**
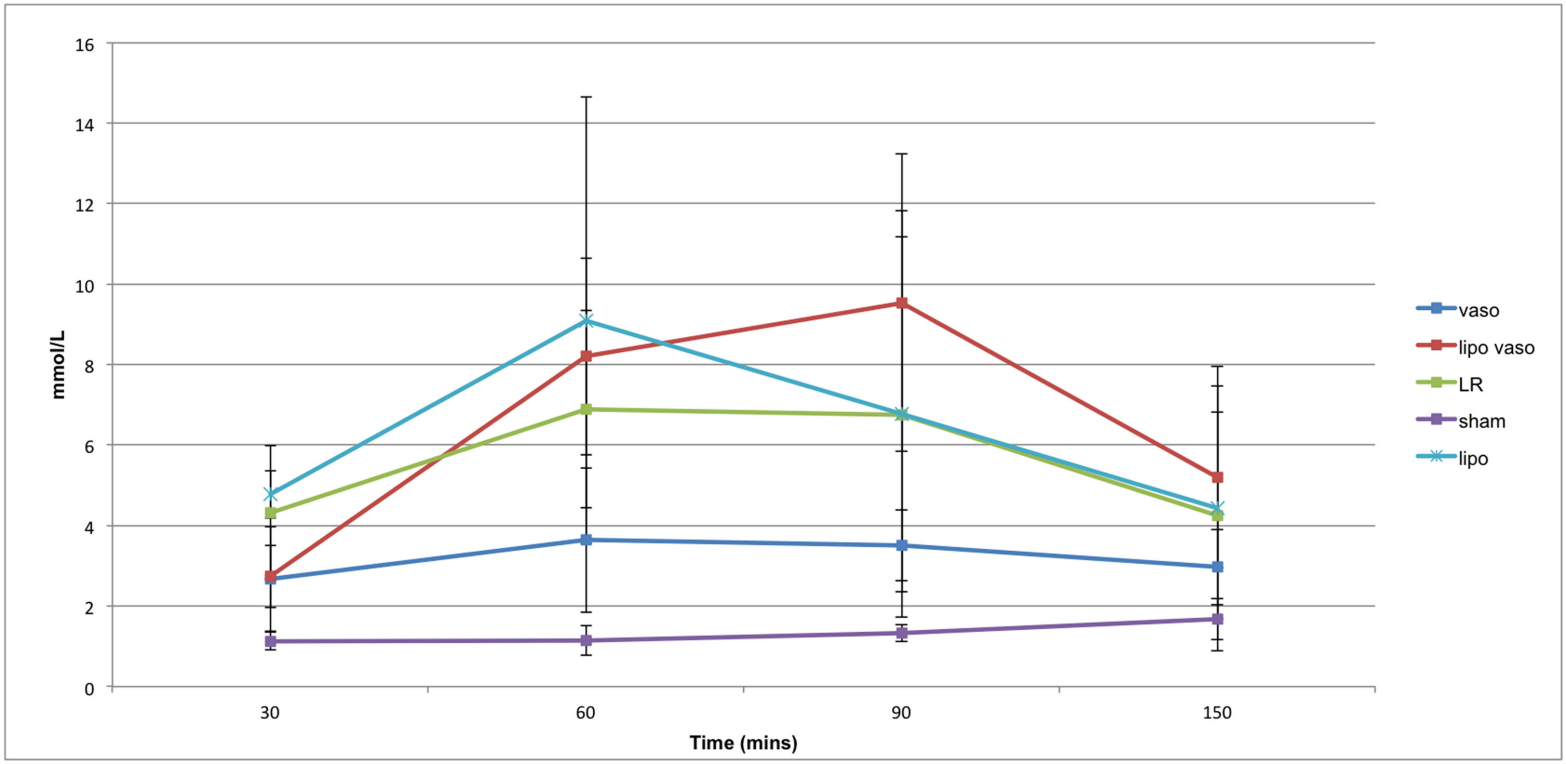
Serum lactate levels for the five treatment groups over time. Vaso group refers to rats treated with vasopressin. Lipo vaso group refers to rats treated with liposomal vasopressin. LR group refers to rats treated with lactated ringer solution without any drug. Sham group refers to rats not undergoing any treatment. Lipo group refers to rats treated with liposome only.

Serial measurements of mean (±SD) serum lactate level versus time plots in the five groups are shown in Fig 3. As expected, the serum lactate levels rose after inducing hemorrhagic shock in all the 4 groups of animals except the Sham group. The vasopressin treated group has a lower serum lactate profile than the liposomal vasopressin treated group (p <0.01). However, there is no significant difference between the serum lactate level in liposomal vasopressin, liposome only and LR treated rats.

### Inflammatory profile

**Fig 4:**
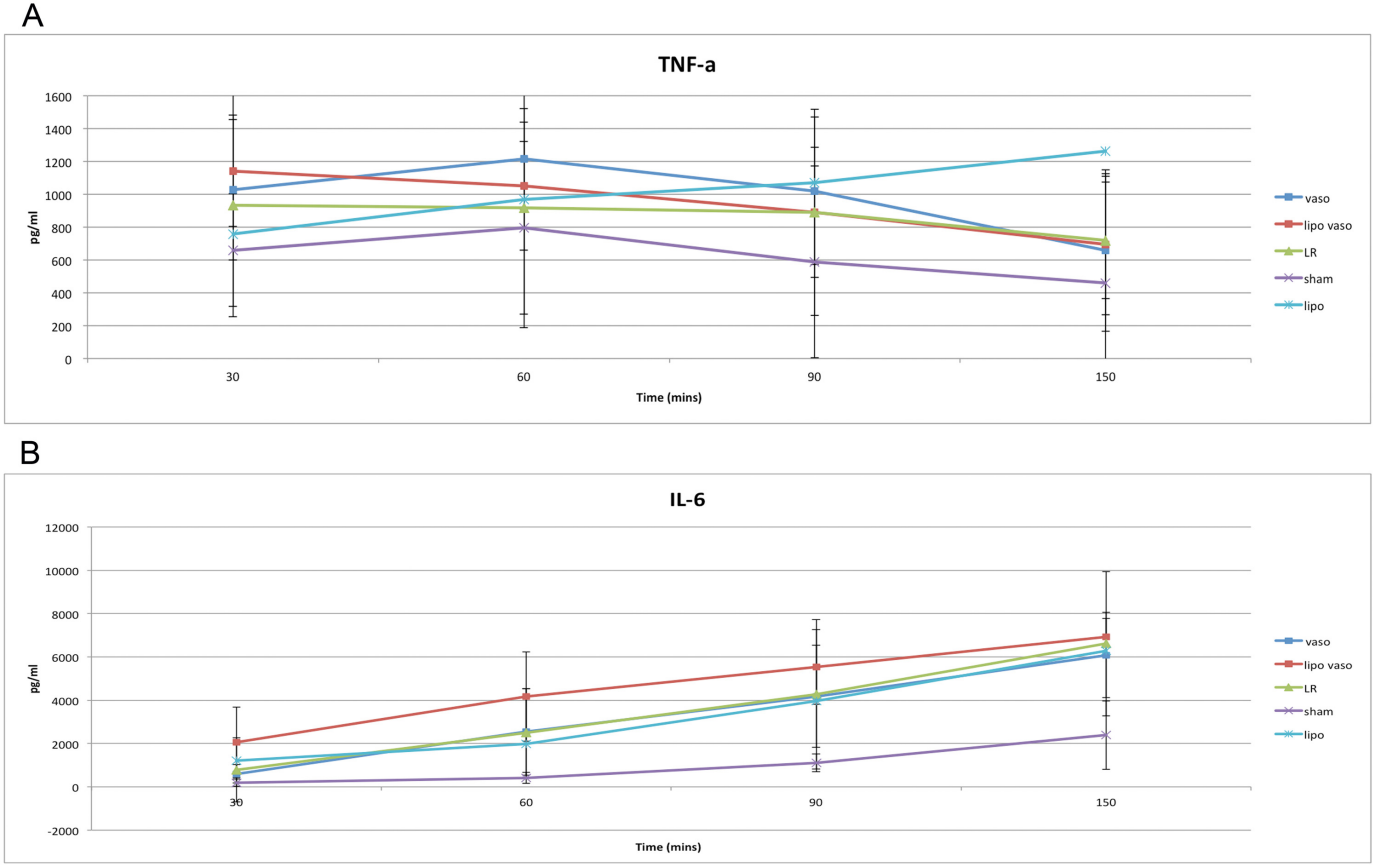
Serum IL-6 (A) and TNF-a (B) level for the different treatment groups. Vaso group refers to rats treated with vasopressin. Lipo vaso group refers to rats treated with liposomal vasopressin. LR group refers to rats treated with lactated ringer solution without any drug. Sham group refers to rats not undergoing any treatment. Lipo group refers to rats treated with liposome only.

Fig 4 shows the inflammatory profile of TNF- a and IL-6 for the different groups over time. During UHS, the TNF-a level in most drug treated groups increase from 30 to 60 minutes and then decreases gradually after 60 minutes till the end of experiment. However, there was no statistical difference in the level of TNF-a in liposomal vasopressin, vasopressin, liposome only and LR treated rats. IL-6 has a slightly different profile than TNF-a. The level of IL-6 in all experimental groups increased from 30 minutes to 150 minutes. Liposomal vasopressin showed a higher level of IL-6 than the other experimental groups. However, there was no statistical difference in the level of IL-6 in liposomal vasopressin, vasopressin, liposome only and LR treated rats.

### Pulmonary edema

**Fig 5:**
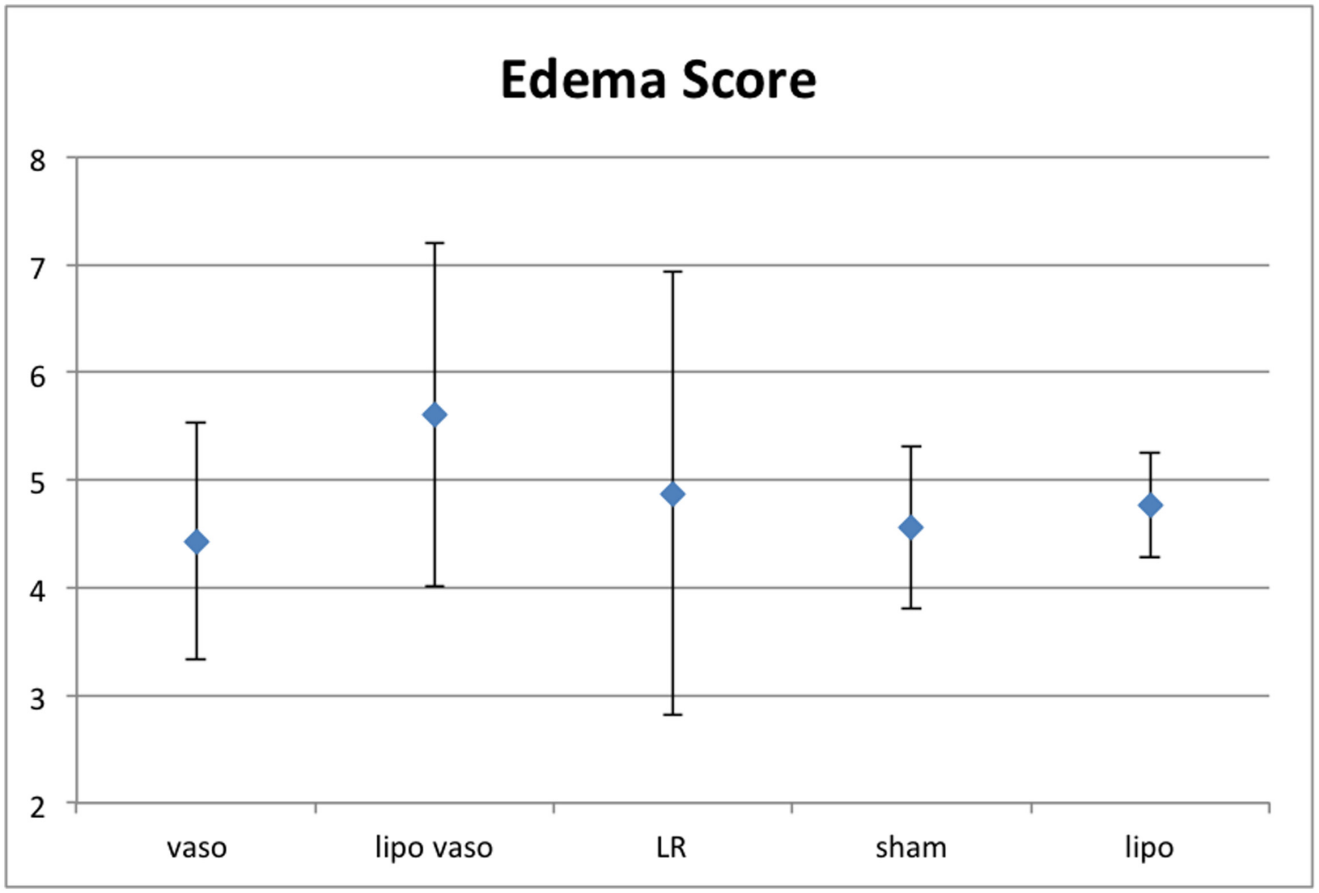
Edema score for the 5 treatment groups. Vaso group refers to rats treated with vasopressin. Lipo vaso group refers to rats treated with liposomal vasopressin. LR group refers to rats treated with lactated ringer solution without any drug. Sham group refers to rats not undergoing any treatment. Lipo group refers to rats treated with liposome only.

UHS can cause an increase in pulmonary edema. In Fig 5, we compared the effect of different drug treatment on pulmonary edema. All the treatment groups have higher pulmonary edema than the sham group, except for the vasopressin treated group. However, there is no statistical significance difference in the pulmonary edema score in liposomal vasopressin, vasopressin, liposome only and LR treated rats.

## Discussion

In this study, we used a film hydration method to prepare for vasopressin encapsulated in the lipids DPPC, cholesterol, and DPPE. Our light scattering data suggested that we had prepared lipid-encapsulated vasopressin with an average vesicle diameter of 535nM. Animals were then subjected to UHS and randomly received 5 different treatments (vasopressin, liposomal vasopressin, lactate ringer, liposome only and sham). The serum concentration of vasopressin was determined at different time points by ELISA. Comparing liposomal vasopressin and vasopressin treated animal at 60 minutes, we found that there is two fold higher serum concentration of vasopressin for the liposomal vasopressin treated group. Outcome of UHS were then measured using 4 common prognostic tests: MAP, serum lactate level, inflammatory profile and pulmonary edema. In three out of four UHS prognostic tests (MAP, inflammatory profile and pulmonary edema), we found that there was no significant difference between rats treated with either vasopressin or liposomal vasopressin. Vasopressin treated animals have significantly better serum lactate level than animals treated with liposomal vasopressin. In addition, we found that the liposome only control behaved similarly to LR resuscitation on MAP, serum lactate level, cytokine profile and edema profile. Although we have not carried out an extensive investigation on the effects of liposome on the immune system and other organs, we hypothesize that liposome administration should be non-immunogenic, biodegradable and non-toxic base on past reports [31, 32].

Even though our results did not agree with our original hypothesis that rats treated with liposomal vasopressin would have better outcome than rats treated with vasopressin, we still made an innovative advance that the scientific community would like to learn more about. Successful demonstration of lipid-encapsulated peptide *in vitro* often does not translate well to the complicated animal system. Our ELISA results at 60 minutes suggest that the lipid-encapsulated vasopressin may be protected from enzymatic degradation or may be acting as a “microreservoir” to release the peptide slowly. However, a higher serum concentration of vasopressin for the liposomal vasopressin group for a prolonged period does not result in a better outcome in our assays. Current hypothesis is that our liposomal vasopressin is either not targeted to the V_1_ receptor, or the free form of vasopressin is not released from the liposome at the correct timing. Vasopressin has three receptors (V_1_, V_2_ and V_3_), but the V_1_ receptor is mainly responsible for vasoconstriction. V_1_ receptor is mainly found in high density on vascular smooth muscle, and there are reports that different composition of liposome can alter content release rate and localization of encapsulated peptide [20, 31, 33–35]. It will be interesting to label either the vasopressin or the lipid to find out if our hypothesis is correct.

In addition, there are reports that efficacy of liposomal drug can be reduced by incorrect targeting to the reticuloendothelial system (RES) system [36, 37]. The vasopressin receptors that are responsible for vasoconstriction are found on the smooth muscles. Uptake of liposomal vasopressin by cells in the RES system can decrease the pool of liposomal vasopressin circulating to the different smooth muscles. Hence, it is attractive to speculate that this is one of the reason why we observed a slight decrease in the level of MAP, when we compare rats treated with liposomal vasopressin and vasopressin.

An optimal liposomal mixture is also required to encapsulate vasopressin in high efficiency *in vitro*, and at the same time allows rapid releases at the right location *in vivo*. Hence, the composition of our liposome mixture was based on our previous experiences, and independent report that high ratio of DPPC and cholesterol can improve the incorporation of the peptide insulin [20, 23–25]. However, different peptides have different properties, and it will be worthwhile to investigate the effects of different lipid composition.

Another limitation is that our small sample (≥4 animal in each experimental group) may be underpowered to show subtle but important differences between vasopressin and liposomal vasopressin treated UHS rats. At a one-sided alpha level of 0.05 and a beta level of 0.2, at least 279 animals in each group are required to identify a difference of 10% in our assays.

In conclusion, our study showed that we have made a functional liposomal vasopressin that can be used in rat model of UHS. However, our liposomal vasopressin did not perform better or might even perform worse than vasopressin in UHS rats. Despite the negative data, it would still be worthwhile to repeat our experiment using different composition of liposome and find out if liposomal vasopressin can be a better therapy for patients that require long traveling time to the trauma center. In the past decade, there are little breakthroughs in hemorrhagic shock resuscitation, and our results will be useful to other researchers who wish to advance this difficult field of research.

## Supporting Information

S1 Fig Concentration of serum vasopressin for the different treatment groups over time. Vaso group refers to rats treated with vasopressin. Lipo vaso group refers to rats treated with liposomal vasopressin. LR group refers to rats treated with lactated ringer solution without any drug. Lipo group refers to rats treated with liposome only.
(TIFF)
