# Supplementary figures and images for: Resuscitation Using Liposomal Vasopressin in an Animal Model of Uncontrolled Hemorrhagic Shock
Source: PLoS One. 2015 Jul 8;10(7):e0130655. doi: 10.1371/journal.pone.0130655 (PMC4496076; doi:10.1371/journal.pone.0130655)

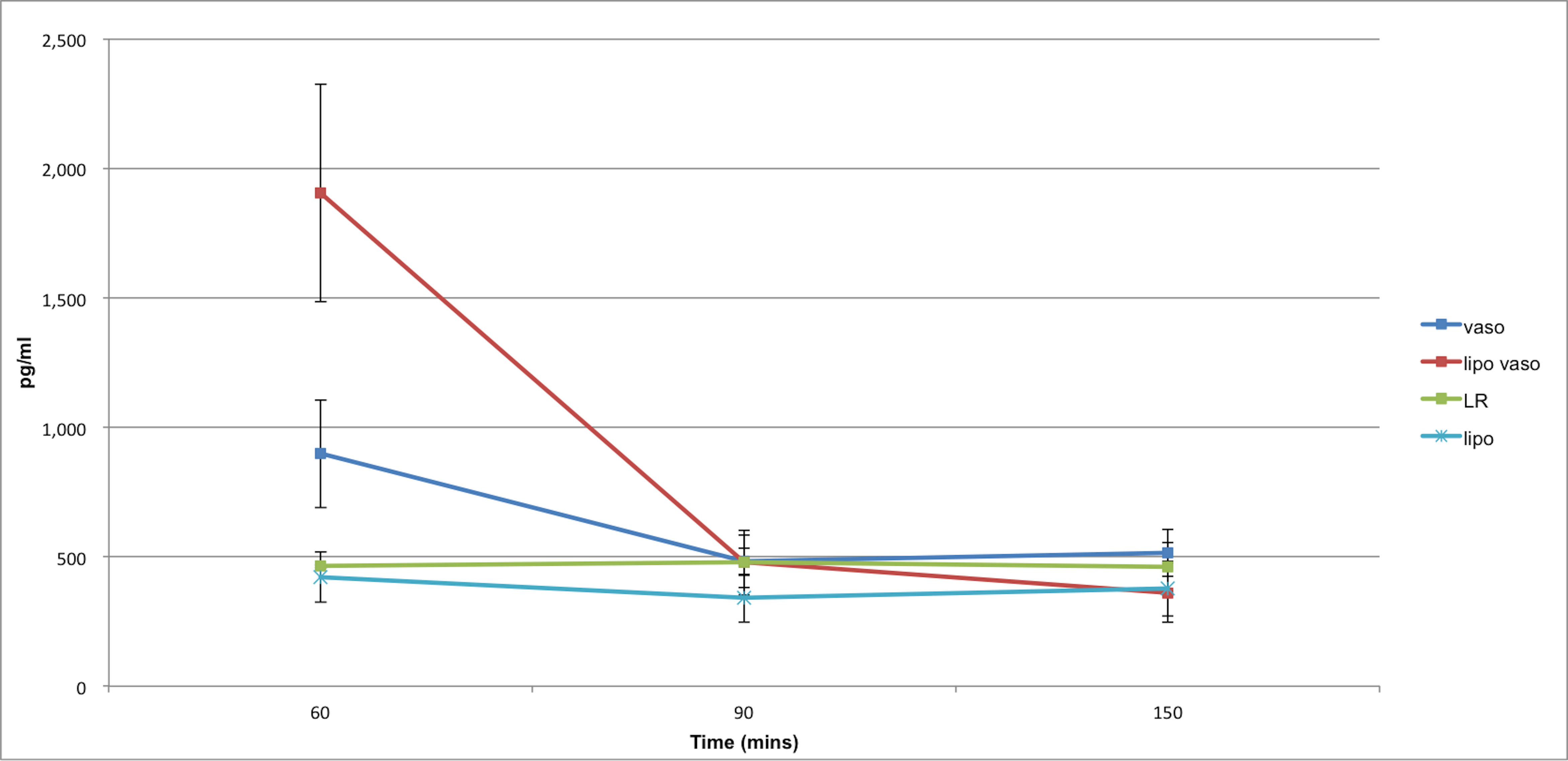

Supplement: S1 Fig — Vaso group refers to rats treated with vasopressin. Lipo vaso group refers to rats treated with liposomal vasopressin. LR group refers to rats treated with lactated ringer solution without any drug. Lipo group refers to rats treated with liposome only. (TIFF) [file pone.0130655.s001.tiff]
